# Pandemics are here to stay: It's time for unity, both nationally and globally, in how we learn and respond

**DOI:** 10.1016/j.clinme.2024.100244

**Published:** 2024-09-20

**Authors:** Ponnusamy Saravanan

**Affiliations:** a Warwick Applied Health, Warwick Medical School, University of Warwick, Coventry, United Kingdom; b Diabetes, Endocrinology and Metabolism, George Eliot Hospital NHS Trust, Nuneaton, United Kingdom

I am delighted to write my editorial for this issue of *Clinical Medicine* following the summer break. I have chosen two articles from this issue to highlight. Lee *et al*^1^ emphasise the importance of recognising the signs and symptoms of measles and conducting appropriate testing to detect it early. With vaccination rates declining and measles becoming more prevalent, the report offers valuable insights and the need for early suspicion and timely intervention. It also underscores the importance of eliminating infections in a country to prevent their resurgence.

While concerns about another potential pandemic, such as Mpox, are looming, the legacy of COVID-19 is far from over. The UK was one of the first to allocate funding for the creation of 90 long COVID clinics, even when understanding of the condition was still developing. Derbyshire *et al*^2^ describe the LOCOMOTION project, which conducted quality improvement work across ten of these clinics. The primary aim is to work collaboratively on this evolving condition, delivering up-to-date, evidence-based care in a multidisciplinary team (MDT) setting. Latest evidence was shared at every MDT, and priority-setting exercises were carried out in collaboration with patient advisory group across these clinics. Though not without challenges, their efforts are commendable and demonstrate that, in the absence of RCT (randomised controlled trial) evidence, such a collaborative approach can be effective. It also highlights the value of working together – with healthcare professionals and patients – when tackling emerging health conditions like long COVID.

Pandemics and their consequences are likely here to stay. It's time we come together to tackle these challenges and minimise their impact on both patients and healthcare professionals.

## Declaration of competing interest

None.
